# *In silico* Druggability Assessment of the NUDIX Hydrolase Protein Family as a Workflow for Target Prioritization

**DOI:** 10.3389/fchem.2020.00443

**Published:** 2020-05-29

**Authors:** Maurice Michel, Evert J. Homan, Elisée Wiita, Kia Pedersen, Ingrid Almlöf, Anna-Lena Gustavsson, Thomas Lundbäck, Thomas Helleday, Ulrika Warpman Berglund

**Affiliations:** ^1^Science for Life Laboratory, Department of Oncology-Pathology, Karolinska Institutet, Stockholm, Sweden; ^2^Chemical Biology Consortium Sweden (CBCS), Science for Life Laboratory, Department of Medical Biochemistry and Biophysics, Karolinska Institutet, Stockholm, Sweden; ^3^Mechanistic Biology and Profiling, Discovery Sciences, R&D, AstraZeneca, Gothenburg, Sweden; ^4^Department of Oncology and Metabolism, Sheffield Cancer Centre, University of Sheffield, Sheffield, United Kingdom

**Keywords:** druggability, nudix, drug discovery, workflow, malachite green

## Abstract

Computational chemistry has now been widely accepted as a useful tool for shortening lead times in early drug discovery. When selecting new potential drug targets, it is important to assess the likelihood of finding suitable starting points for lead generation before pursuing costly high-throughput screening campaigns. By exploiting available high-resolution crystal structures, an *in silico* druggability assessment can facilitate the decision of whether, and in cases where several protein family members exist, which of these to pursue experimentally. Many of the algorithms and software suites commonly applied for *in silico* druggability assessment are complex, technically challenging and not always user-friendly. Here we applied the intuitive open access servers of DoGSite, FTMap and CryptoSite to comprehensively predict ligand binding pockets, druggability scores and conformationally active regions of the NUDIX protein family. In parallel we analyzed potential ligand binding sites, their druggability and pocket parameter using Schrödinger's SiteMap. Then an *in silico* docking cascade of a subset of the ZINC FragNow library using the Glide docking program was performed to assess identified pockets for large-scale small-molecule binding. Subsequently, this initial dual ranking of druggable sites within the NUDIX protein family was benchmarked against experimental hit rates obtained both in-house and by others from traditional biochemical and fragment screening campaigns. The observed correlation suggests that the presented user-friendly workflow of a dual parallel *in silico* druggability assessment is applicable as a standalone method for decision on target prioritization and exclusion in future screening campaigns.

## Introduction

**Figure 1:**
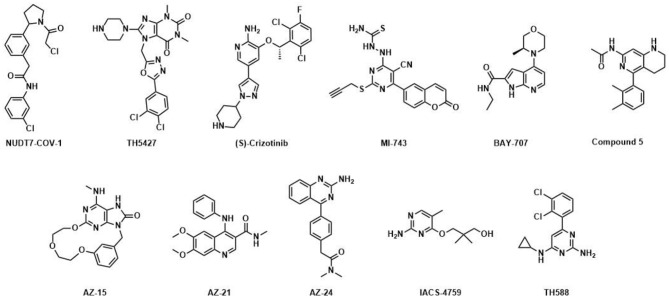
Published NUDIX inhibitors: TH588 was developed as a first in class NUDT1 inhibitor at Science for Life Laboratory and Karolinska Institutet (Gad et al., 2014); (S)-Crizotinib is a potent NUDT1 inhibitor and the enantiomer of (R)-Crizotinib (Huber et al., 2014), a clinically applied tyrosine kinase inhibitor; optimized by Astra-Zeneca; AZ-15, AZ-21 and AZ-24 are distinct chemotype inhibitors targeting NUDT1 (Kettle et al., 2016); BAY-707 (Ellermann et al., 2017) was discovered as a NUTD1 inhibitor by Sprint Bioscience; IACS-4759 (Petrocchi et al., 2016) is a NUDT1 inhibitor developed by MD Anderson; MI-743 is a selective inhibitor of NUDT1 in gastric cancers (Zhou et al., 2019); Compound 5 was reported by Gilead and inhibits NUDT1 (Farand et al., 2020); TH5427 was synthesized as a lead compound against NUDT5 (Page et al., 2018); NUDT7-COV-1 is a covalent inhibitor generated by electrophile screening and fragment combination (Resnick et al., 2019).

The nucleoside diphosphates attached to sequence-x (NUDIX) hydrolase protein family was recently comprehensively and exhaustively reviewed by Carreras-Puigvert et al. (2017) NUDIX proteins possess a conserved sequence, called the NUDIX box, i.e., Gx_5_Ex_5_[UA]xREx_2_EExGU), which differs little between individual members which are otherwise of low sequence similarity. Structural and domain analysis revealed three major groups and one outlier, NUDT22, mostly based on their already reported activity against substrate classes such as diphosphoinositol polyphosphates (Caffrey et al., 1999, 2000) and NADH diphosphates (Abdelraheim et al., 2003). Subsequently, a systematical screening against a large set of substrates was performed and painted a rather promiscuous picture of the NUDIX hydrolases, indicating backup functionality or redundancy. Consequently, a global expression analysis was performed and showed a clear dependency on tissue of origin and the corresponding cancer tissue. Interestingly, NUDT1, NUDT5, and NUDT14 amongst others were present in a cluster of highly expressed proteins, confirming a potential role in cancer as reported earlier (Choi et al., 2011; Gad et al., 2014; Huber et al., 2014; Wright et al., 2016). Importantly, when evaluated for epistasis, it became apparent that several NUDIX members sustain relations as measured in cell viability and cell cycle perturbations and that these interactions are more important for cancerous cells. With this overview in structure, expression, substrate specificity and relation, the NUDIX protein family members gained considerable attention as potential drug targets. The original interest in pharmacological modulation of NUDIX members was sparked by the notion that NUDT1 is overexpressed in several cancer cell types, while its role in healthy cells can largely be compensated for as evidenced by the normal life-span of knock-out mice (Tsuzuki et al., 2001). Besides GTP and dGTP, NUDT1 hydrolyzes several oxidatively damaged DNA nucleotides including 8-oxo-dGTP and 2-OH-dATP, thus preventing their incorporation into DNA, which otherwise would lead to DNA damage and ultimately cell death. This led to the hypothesis that increased expression of NUDT1, and hence improved sanitization capacity of oxidatively damaged DNA bases from the nucleotide pool, would enable cancer cells to cope with the increased oxidative stress they are exposed to compared with healthy cells. Gad and coworkers published TH588 (Figure 1) as the first small-molecule NUDT1 inhibitor with efficacy in mouse xenograft models (Gad et al., 2014), although subsequent potent and selective NUDT1 inhibitors disclosed by AstraZeneca, MD Anderson, Gilead and Sprint Bioscience/Bayer failed to reproduce these findings with regards to cytotoxicity (Figure 1) (Kettle et al., 2016; Petrocchi et al., 2016; Ellermann et al., 2017; Farand et al., 2020). The validity of NUDT1 as an anticancer target has thus been questioned and is still under debate (Warpman Berglund et al., 2016; Samaranayake et al., 2017). Regardless, these studies served to demonstrate significant amenability to small-molecule inhibition of NUDT1, justifying the question as to how this translates to other members of the NUDIX family.

Besides NUDT1, a series of potent, drug-like NUDT5 inhibitors have been described by Page and coworkers (Page et al., 2018). The lead compound TH5427 (Figure 1) was shown to block progestin-dependent, PAR-derived nuclear ATP synthesis and subsequent chromatin remodeling, gene regulation and proliferation in breast cancer cells, suggesting that targeting NUDT5 may represent a novel therapeutic approach for breast cancer treatment. Most recently, the covalent NUDT7 inhibitor NUDT7-COV-1 was developed by employing electrophilic fragment screening and a fragment combination approach (Figure 1) (Resnick et al., 2019). To the best of our knowledge no potent inhibitors for any of the other NUDIX hydrolase members have been disclosed to date, although there are public data on hit rates for selected family members on the Structural Genomics Consortium homepage^1^^,^ ^2^.

One aspect not addressed in the recent comprehensive review (Carreras-Puigvert et al., 2017) is an assessment of the potential druggability of the different NUDIX family members, i.e., their amenability to be modulated by drug-like small molecules. With the recent dawn of PROTACs, synthetic neoantigens and biologics, but also established targeting strategies like allosteric modulation or active site inhibition, several scenarios of how to target a protein may be exploited. With that in mind, druggability as such is no longer restricted to active site inhibition of a protein by a small molecule with an optimized small-molecule drug-like profile. Both orthosteric or catalytic sites and secondary, allosteric sites, may be equally interesting to be targeted for the development of small-molecule chemical probes and potential drug candidates. As high-resolution crystal structures of 18 out of the 22 human NUDIX hydrolases are now available, a family-wide *in silico* druggability assessment for available sites is feasible.

Here we use several open-access binding site analysis methods, i.e., DoGSite (Volkamer et al., 2012)^3^, CryptoSite (Cimermancic et al., 2016)^4^, and FTMap (Kozakov et al., 2015)^5^, as well as the commercial SiteMap and *in silico* fragment screening of a fragment library using Glide to probe the NUDIX hydrolase protein structures for potential small-molecule binding sites and assess their druggability and suitability for a prospective drug discovery campaign. This established *in silico* prioritization workflow within the NUDIX family is further supported by results obtained from biochemical screens employing the malachite green assay (Baykov et al., 1988) as well as differential scanning fluorimetry (DSF) (Niesen et al., 2007) fragment screens for some of the family members. This correlation with own experimental results and those published previously highlights the benefit of this comparably low-cost computational assessment workflow prior to applying experimental screening methods for the rapid evaluation of target druggability.

## Materials and Methods

### Protein Preparation and Validation

Available crystal structures of human NUDIX hydrolases with the highest resolution were imported into Maestro (Schrödinger Suite 2019-1, Schrödinger, LLC, New York, NY, 2019.) The structures were then prepared using the Protein Preparation Wizard as implemented in the Schrödinger Suite. Briefly, raw PDB structures were processed by automatically assigning bond orders, adding hydrogens, creating zero-order bonds to metals, converting selenomethionine to methionine, adding missing side-chains, creating possible disulfide bridges, deleting waters beyond 5.0 Å of hetero groups (if present), and generating hetero protonation states at pH 7.0. Residues with alternate positions were locked in the conformations with the highest average occupancy. Small ligands and metal ions originating from crystallization buffer were removed. The hydrogen bonding networks were optimized automatically, by sampling water orientations and optimization of hydroxyls, Asn, Gln, and His residue states using ProtAssign. Any remaining water molecules were subsequently removed. A restrained minimization was then performed using the OPLS3e force field, until an RMSD convergence of 0.30 Å was reached for the heavy atoms. Finally, the minimized NUDIX structures were aligned to the structure of NUDT1 (3Q93) with respect to the backbone atoms of the A chain.

### DoGSite

The protein structures as prepared above were exported as PDB files, uploaded to the DoGSite server and assessed for binding sites and their corresponding DrugScores according to the published protocol (Volkamer et al., 2012). Pocket Size and DrugScores were extracted for all identified sites and annotated to pocket numbers.

### FTMap

All prepared PDB files were uploaded to the FTMap server and interrogated for number of probes per cluster found according to the published protocols (Kozakov et al., 2015; Vajda et al., 2018).

### CryptoSite

All prepared PDB files were uploaded to CryptoSite server and assessed for amino acid flexibility according to the published protocol (Cimermancic et al., 2016). Amino acid residues exceeding a Cryptic Site Score of 0.10 were extracted.

### SiteMap

Prepared protein structures were submitted to SiteMap analyses as implemented in Schrödinger Suite 2019-1. The 5 top-ranked potential binding sites were identified. At least 15 site points per reported sites were required. The more restricted definition of hydrophobicity together with a standard grid (0.7 Å) were used. Site maps at 4 Å or more from the nearest site points were cropped. Clustering of the SiteMap parameters was performed using the heatmaply library in R^6^. The SiteMap parameters were transformed using “percentize,” and average linking was used for clustering.

### Virtual Fragment Screening

1) Fragment subset selection: a subset of the ZINC Frags Now set (Irwin et al., 2012) was created by applying a number of filters implemented in a Knime workflow (Knime 3.5.2, Berthold et al., 2008). Foremost, only fragments available from a list of 19 preferred suppliers, composed by a team of experienced medicinal chemists were considered. These were then filtered using a cascade of structural filters, including REOS (Walters and Murcko, 2002), PAINS (Baell and Holloway, 2010) and a set of in-house filters (ScrapFilter) compiled over the years. Lipinski-type descriptors (SlogP, TPSA, AMW, NumLipinskiHBA, NumLipinskiHBD, NumRotatableBonds, NumHeavyAtoms, NumRings, NumAromaticRings) were then calculated using the RDKit Descriptor Calculation node. An additional descriptor HetRatio was then calculated as the ratio of NumLipinskiHBA and NumHeavyAtoms, and fragments with HetRatio <0.2 or >0.5 were filtered out. Finally, remaining outliers were removed by applying Gaussian Z-score normalization on the descriptor space, and then filtering out fragments with descriptor values deviating more than 3 units from the mean. The entire filtering cascade reduced the original input file of 704,041 structures as downloaded from ZINC to 205,891 fragments (Supplementary Data Sheet 1).
2) Ligand preparation: the selected fragment subset was then prepared for docking using LigPrep (Schrödinger): the OPLS3e force field was used for minimizations; possible ionization states at pH 7.0 ± 2.0 were generated using Epik (Shelley et al., 2007; Greenwood et al., 2010), metal binding states were added, and tautomers were generated; specified chiralities were retained and at most 4 stereoisomers were generated per structure. This yielded 345,044 structures for docking.
3) Receptor grid generation: Glide docking grids (Friesner et al., 2004, 2006; Halgren et al., 2004) were generated for each target protein by focusing the grid box on the center of the site with the highest Dscore as determined by SiteMap (Halgren, 2007, 2009). The size of the box enclosing the grid was set to 16 Å. No constraints, rotatable groups or excluded volumes were defined.
4) Virtual screening: The Virtual Screening Workflow as implemented in Schrödinger Suite was used for docking, scoring, and ranking of the top-1,000 fragments against the sites with the highest Dscore as determined by SiteMap. The workflow comprised a cascade of docking steps with increased accuracy (Glide HTVS → SP → XP), where the top-10% ranked ligands are passed on to the next step. After Glide XP docking the top-1,000 ranked fragments were retained for druggability assessment based on their combined docking scores.

### Biochemical Screening

Small-molecule screening of NUDT2, NUDT15, and NUDT16 at a compound concentration of 10 μM was conducted using coupled enzymatic assays as already described for NUDT1 (Gad et al., 2014) and NUDT5 (Page et al., 2018). In brief this involved the purification of recombinant proteins following overexpression in *E. coli* and subsequent validation of coupled enzymatic assays based on cognate substrates for each of these [Ap4A for NUDT2, dGTP for NUDT15 and ADP for NUDT16 (Trésaugues et al., 2015)]^2^. The assays for NUDT2 and NUDT15 were based on enzymatic release of inorganic pyrophosphate and subsequent degradation to two molecules of inorganic phosphate in the presence of excess inorganic pyrophosphatase. Levels of inorganic phosphate are measured using an established procedure for such measurements in 384-well format in our lab (see e.g., Gad et al., 2014; Page et al., 2018). The screening of NUDT16 was based on enzymatic processing of ADP to release one molecule of inorganic phosphate, such that the coupled enzyme was not needed in this assay. All assays were optimized to allow their application at close to the K_m_ of each substrate and with an incubation time chosen to ensure consumption of <30% of substrate and near linearity of assay signal increase with time.

Slightly different screening sets have been applied for the family members, with only a smaller subset of 5,500 compounds in common. All screens conducted at Chemical Biology Consortium Sweden have 16 each of negative (DMSO only – 0% inhibition) and positive controls (no enzyme or inhibitor at concentration that gives 100% inhibition). These are located in columns 23 and 24 of the 384-well plates and they are used to normalize the response in each well-containing library compounds to a % inhibition value. Hit limits are defined based on the average plus three standard deviations of the response for all library compounds and hit rates are provided as the percentage of library compounds above this limit. The malachite green assay has been extensively used for screening purposes in our lab as it is associated with low interference rates, as evidenced by the lack of common hits appearing in screens of NUDT1 (Gad et al., 2014), NUDT5 (Page et al., 2018), dCTPase (Llona-Minguez et al., 2016), dUTPase and ITPase besides the herein reported NUDIX proteins (Supplementary Material – Screens using malachite green).

### DSF Fragment Screening

NUDT1, NUDT2, NUDT5, and NUDT15 druggability was further experimentally assessed through fragment screening by DSF. Different fragments sets were screened over time, reflecting history and development of the available fragment sets. The initial fragment library comprised 450 fragments selected from the Chemical Biology Consortium Sweden reagent store at the Karolinska Institutet, and this set was screened against NUDT1 and NUDT5. Over time this library was complemented with sets of nucleobase analogs acquired from the NCI Developmental Therapeutics Program, which was grown to a subset of 200 compounds. This set, together with the 450-member library, thus totaling 650 fragments, was screened against NUDT2. Subsequently the 450-member library was complemented with 550 additional fragments from the Chemical Biology Consortium Sweden reagent store in order to generate a more diverse generic fragment library of 1,000 compounds. This second version together with the 200 nucleobase analogs acquired from NCI was screened against NUDT15. The proteins were expressed and purified as previously reported (Carreras-Puigvert et al., 2017). Fragment screening by DSF was essentially performed as described in detail by Niesen et al. (2007) All fragments were screened at a final concentration of 500 μM. Positive controls for each target were used at 100 μM. Assay buffer was composed of 100 mM Tris Acetate, 40 mM NaCl, and 10 mM Mg Acetate. Sypro Orange (S6650, Molecular Probes, 5000x) was used as the fluorescent dye. Native melting points of the proteins under the assay conditions were 50.0, 50.0, 76.0, and 57.0°C for NUDT1, NUDT2, NUDT5, and NUDT15, respectively. Screening was performed in 96-well Q-PCR plates using a BioRad 96CFX real-time PCR detection system with temperature increments of 1.0°C. More details of the assay conditions for each target are provided in the Supplementary Material – Fragment screen conditions.

## Results and Discussion

### Automated Arm - Step 1: DoGSite and FTMap Predict Druggable Catalytic Sites and Potentially Druggable Secondary Sites

**Figure 2:**
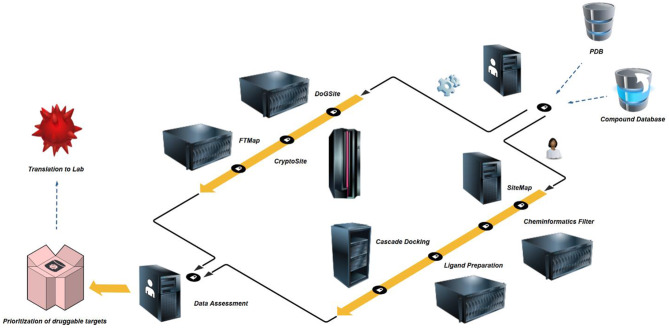
*In silico* druggability prioritization workflow – High-resolution crystal structures and comprehensive commercial space compound databases are freely available. PDBs are prepared by protein preparation wizard (Thorsell et al., 2009), inspected for structural flaws and limitations and exported as new structure files. In a first step, the structures are assessed for binding sites and site druggability by algorithms using rigid proteins or allowing for flexible behavior, i.e. DoGSite (Volkamer et al., 2012) and CryptoSite (Cimermancic et al., 2016). To eliminate artifacts due to protein construct choice, limitations of crystal structure resolution or co-crystallization of additional protein copies, all identified sites are counter-screened by FTMap (Kozakov et al., 2015). In a second and parallel step, the same structures are initially assessed for binding sites, druggability and pocket parameter using SiteMap (Halgren, 2007, 2009). A separate Knime (Berthold et al., 2008) workflow for the elimination of promiscuous functionalities is followed by Ligand Preparation which builds an applicable set of small molecules including a number of tautomers and stereoisomers. In a three-step cascade this set is then docked (Friesner et al., 2004, 2006; Halgren et al., 2004) against the highest-ranking site as identified by SiteMap. The median docking score of the top-1,000 fragments is used to assess druggability based on commercial fragment space. In a final step, prioritization of targets passing both parallel screening schemes may be performed based on published experimental screening data or own future screening efforts during translation to the lab.

We started by compiling a list of available high-resolution crystal structures of human NUDIX proteins (Table 1). Due to the systematic work of the Structural Genomics Consortium, the majority of structures were solved with high sequence coverage (Supplementary Material – SiteMap secondary sites) and are often available together with screening data^1^. PDBs were imported to Maestro and prepared as described in the Method part. To enable application in the automated workflow, the prepared proteins were exported as new PDB files (Figure 2). In a first step, these files were uploaded to the DoGSite server. DoGSite is a web-based open-access algorithm that interrogates rigid protein structures for binding hotspots, including druggability prediction (Volkamer et al., 2012). Initially, a grid covering the protein identifies grid points that overlap with protein atoms. Application of a difference of Gaussian (DoG) filter then screens for preferred binding spots of sphere-like objects. Combination of several hotspots creates subpockets, which, if neighboring, are merged into a pocket. Several geometric and physico-chemical properties are automatically calculated for the predicted pockets and subpockets. A machine learning model trained on a set of known druggable proteins is then used to predict the druggability of the pockets, expressed as DrugScore. Reported as a factor between 0 and 1.0 a DrugScore over 0.5 and closer to 1.0 corresponds to good druggability.

**Table 1:** High-resolution crystal structures used in this study.

**Protein name**	**PDB code**	**References**
NUDT1, MTH1	3Q93	Tresaugues et al., 2011a
NUDT2, APAH1	3U53	Ge et al., 2013
NUDT3, DIPP1	2FVV	Thorsell et al., 2009
NUDT4, DIPP2	5LTU	Srikannathasan et al., 2017a
NUDT5, HSPC115	6GRU	Dubianok et al., 2018
NUDT6, FGF2AS	3H95	Tresaugues et al., 2009a
NUDT7	5T3P	Srikannathasan et al., 2017b
NUDT9	1Q33	Shen et al., 2003
NUDT10, DIPP3A	3MCF	Tresaugues et al., 2010
NUDT12	6SCX	Wu et al., 2019
NUDT14, UGPP	3Q91	Tresaugues et al., 2011b
NUDT15, MTH2	5BON	Carter et al., 2015
NUDT16	3COU	Tresaugues et al., 2008
NUDT17	5LF8	Mathea et al., 2017a
NUDT18, MTH3	3GG6	Tresaugues et al., 2009b
NUDT20, DCP2	5MP0	Mathea et al., 2017b
NUDT21	3BAP	Coseno et al., 2008
NUDT22	5LF9	Tallant et al., 2017
PTP1B^*^	2HNP	Barford et al., 1994

* *Added as reference protein*.

**Figure 3:**
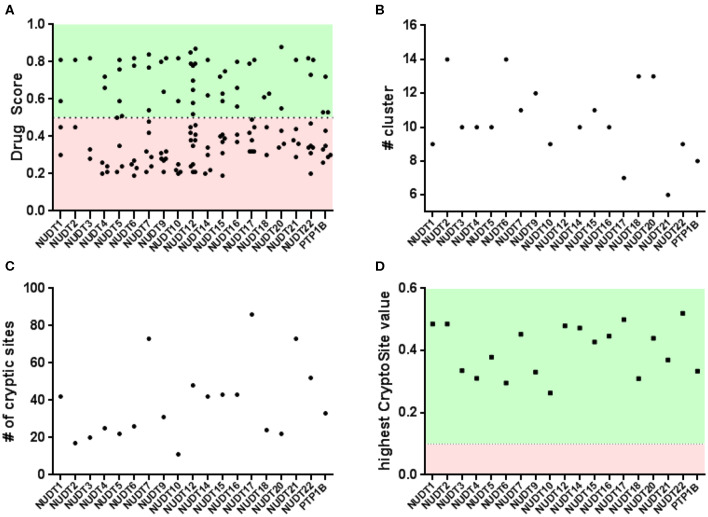
Binding pocket identification and druggability assessment in the automated arm: **(A)** rigid protein assessment with DogSite identifies a number of binding sites within a range of predicted druggability scores; **(B)** FTMap identifies a number of cluster per protein and only NUDT4 and NUDT18 fail to contain enough samples per cluster in the active site; **(C,D)** flexible protein assessment with CryptoSite identifies amino acid residues with different degrees of conformational freedom. Including the top scoring residues these networks largely overlap with pockets identified by DogSite.

Application of this algorithm to NUDIX crystal structures identified between two and ten pockets with a wide range of DrugScores (Figure 3 and Supplementary Material - DogSite). Between one and four bindings sites were judged druggable by the DoGSite algorithm. For some of the NUDIX hydrolases the natural substrates and their binding sites are yet to be deciphered. In addition, with the broad targeting possibilities provided by PROTACs (An and Fu, 2018) or allosteric inhibitors (Wenthur et al., 2014; Aretz et al., 2018), it is not necessarily required to target a catalytic pocket to convey a desired phenotype. Thus, the single highest-ranking site of each NUDIX structure, often corresponding to the known substrate binding site, was used to calculate a NUDIX druggability score. With an average druggability score of 0.80, the NUDIX family of proteins qualify as good predicted drug targets. As a positive control and validated target when it comes to chemical amenability, NUDT1 (3Q93) reaches a similar score of 0.81. The protein tyrosine phosphatase 1B (PTP1B) was included into the assessment (2HNP) as this is generally known to be a challenging target for classical drug discovery approaches. PTP1B, like other tyrosine phosphatases, contains a relatively polar substrate pocket which can accommodate phosphate isosteres. In the last two decades, small molecules targeting this pocket have been shown to fail eliciting sufficient effects *in vivo* (Zhang and Zhang, 2007; Krishnan et al., 2018). Instead a non-classical approach of allosteric inhibition is currently under evaluation in clinical trials (Mullard, 2018). When interrogated with DoGSite, PTP1B (2HNP) scores 0.72 only by combination of two subpockets through a narrow channel.

**Figure 4:**
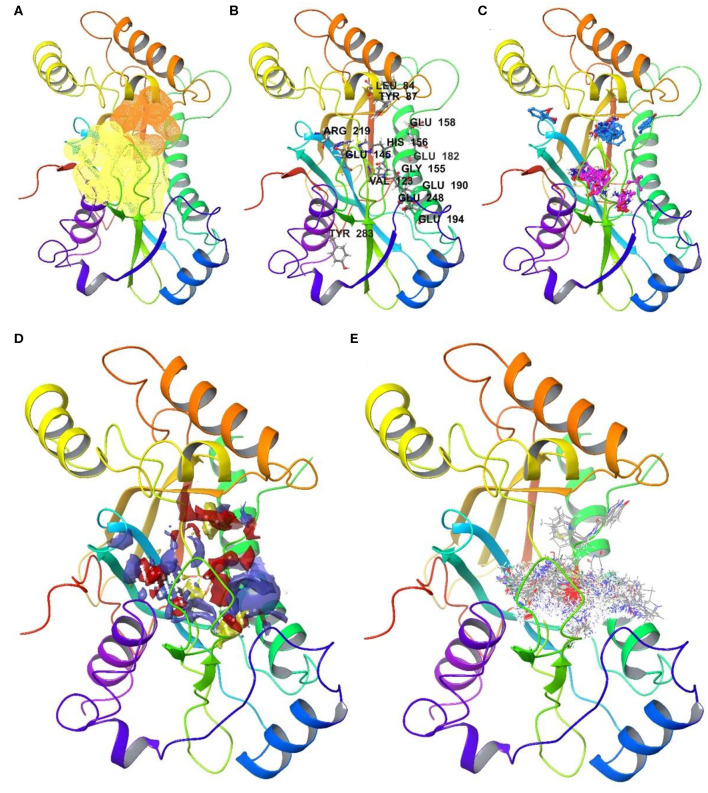
Druggable sites of NUDT22 as identified in the automated and the user arm: **(A)** Rigid assessment with DoGSite identifies a second highly druggable site (orange, DrugScore 0.81) in close proximity to the highest-ranking site (yellow, DrugScore 0.82); **(B)** Flexible assessment with CryptoSite predicts 52 amino acid residues around both pockets as part of an extended 3D network. Highlighted amino acid residues possess cryptic score over 0.2; **(C)** FTMap confirms all cluster hotspots and at least 16 probes within 5 Å radius of either of the binding sites (site 1: pink; site 2: light blue) or high cryptic value sites. **(D)** SiteMap combines the two sites identified by DogSite into a single large binding pocket with an evenly distribution of hydrophobic (yellow) and hydrophilic (red and purple) patches; **(E)** cascade docking of the ZINC fragment library shows a preference for the active site, while the second druggable site is only engaged by members of one chemotype among the top 1000 fragments. The assessment highlights NUDT22 comprising two adjacent druggable sites which in a prospective drug discovery campaign could be targeted separately or in combination.

An interesting observation is that all NUDIX members, except NUDT4 (0.72, 5LTU) and NUDT18 (0.63, 3GG6), individually score a high DrugScore around 0.80. Furthermore, it can be observed that several members, including NUDT6 (3H95, 0.78), NUDT7 (5T3P, 0.77), NUDT9 (1Q33, 0.82), NUDT17 (5LF8, 0.79), NUDT12 (6SCX, 0.85), and NUDT22 (5LF9, 0.81, Figure 4), are predicted to possess a second high-ranking pocket. These sites may increase the potential for pharmacological targeting of the corresponding proteins, for instance by masking a protein-protein interaction or a cofactor binding site. Identification of a second high-ranking pocket remote from the catalytic site, however, may also point toward an artifact in the crystal structure due to the construct used for expression or lack of electron density. For a comparison of resolved and expressed sequences please refer to Supplementary Material – SiteMap secondary sites. Thus, when inspected for their location, it became apparent that secondary sites can be distinguished as either neighboring to the top-ranked site or being located more remotely.

The druggability of the identified pockets can be further assessed using FTMap (Kozakov et al., 2015; Yueh et al., 2019). FTMap interrogates the protein surface for contributions to ligand-free energy. Small organic molecules, reflecting the complexity of potential active substances, are scored using a detailed energy function. Some regions bind several clusters of probes and thus identify as a binding hotspot. Earlier, this orthogonal method was applied on pockets identified by CryptoSite (Vajda et al., 2018), where high druggability would correspond to an FTMap cluster populating these sites and containing at least 16 probes. When similarly examined for the number of bound probes, all highest-ranking sites of each NUDIX protein except for NUDT4 (5LTU) and NUDT18 (3GG6), reached more than 16 probes confirming the good druggability of the expected active sites of the enzyme family (Figure 3 and Supplementary Material - FTMap). NUDT4 (5LTU) and NUDT18 (3GG6), which showed a lower DrugScore before, failed to contain more than 16 probes and are the only family members with a lower druggability assessment based on DoGSite and FTMap. Assessment of PTP1B (2HNP) returned all FTMap probe clusters to be located in the smaller of the two sites predicted by DoGSite (DrugScore 0.38). When evaluated with FTMap, secondary sites of NUDT6 (3H95), NUDT7 (5T3P), NUDT9 (1Q33), NUDT17 (5LF8), and NUDT22 (5LF9, Figure 4) neighboring the highest-ranking site tend to harbor more probes than those sites found remotely. All remote secondary sites, i.e., NUDT9 (1Q33), NUDT17 (5LF8) and NUDT 12 (6SCX), fail to incorporate the required 16 probes. Of those located much closer to the highest-ranking pocket, only NUDT7 (5T3P) fails to accommodate 16 or more probes underscoring the potential use in pharmacological targeting additionally to the neighboring highest-ranking pocket.

### Automated Arm – Step 2: CryptoSite and FTMap Confirm Druggable Active Binding Pockets With High Flexibility

Druggability predictions using DoGSite are based on rigid protein structures, not allowing for flexibility typically induced by larger natural substrates or specifically designed small molecules (Michel et al., 2019). Another aspect is the potential existence of allosteric sites. Typically, a crystal structure of a compound bound to the allosteric site or comprehensive protein dynamics calculations based on several distinct crystal structures are required for their discovery. The CryptoSite algorithm however, can give first insights in whether an already identified active site or a shallow pocket allows for high single amino acid flexibility (Cimermancic et al., 2016). Networks of these flexible cryptic sites could indicate concerted movements of the protein, possibly forming an allosteric site or conformational changes relevant for substrate binding and protein function. Cryptic scores returned by the algorithm above 0.10 and higher consider a site as cryptic and thus flexible.

When interrogated with CryptoSite, NUDIX hydrolases showed an increased number of cryptic sites around the highest-ranking site as identified before by DoGSite, indicating an extended and flexible three-dimensional network of amino acid residues (Figure 3 and Supplementary Material - CryptoSite). Between 11 and 86 and on average 40 residues scored higher than 0.10 (NUDT1, 42; PTP1B, 33). The highest scoring residues reached values between 0.26 and 0.52 and on average 0.40 (NUDT1, 0.49; PTP1B, 0.33). NUDT10 failed to form a cryptic network while NUDT6, NUDT7 and NUDT22 (Figure 4) possessed a second cluster of cryptic sites overlapping with the second highest-ranking sites as identified by DoGSite. Except for NUDT4 (5LTU) and NUDT18 (3GG6), all cryptic networks of the protein family members were populated by more than 16 probes in FTMap (Figure 3 and Supplementary Material – FTMap).

The result of this initial druggability assessment suggest that NUDIX hydrolases are on average good drug targets with regard to their expected or known active sites. Further, only a few members of the family possess a second druggable site as based on DoGSite and FTMap analyses, and even fewer exhibit conformational flexible sites remote from the identified active site.

### User Arm – Step 1: SiteMap Binding Site Prediction and Druggability Assessment

In a second parallel approach we assessed druggability using SiteMap and a Glide-based virtual screening workflow applied to a KNIME filtered fragment library (Figure 2). SiteMap, an application to identify binding pockets and predict druggability, is implemented in the Schrödinger small-molecule modeling suite. Binding pockets identified on the protein surface are given a score, the Dscore, which is based on pocket parameters such as size, exposure to solvent, enclosure by protein, ratio of hydrogen bond donors and acceptors and importantly hydrophilicity, hydrophobicity and a determined ratio thereof. This druggability score favors proteins with a higher hydrophobic/hydrophilic ratio and thus allows for an early assessment of pocket polarity as required for binding of small-molecule drugs. Typical Dscores for druggable protein pockets are above 1.108 while Dscores below 0.871 suggest a difficult to drug protein (Halgren, 2007, 2009). In addition, comparing individual pocket parameters allows for a detailed picture of druggability and for specific assessment of proteins with similar Dscores and/or sequence.

**Figure 5:**
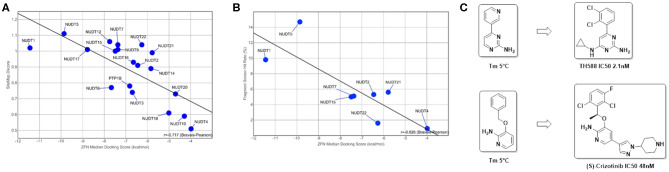
Predictive value of *in silico* assessment and docking for in vitro screening hit rates and suitability of fragment screens for chemical probe generation: **(A)** *in silico* druggability assessment of pockets identified by SiteMap correlates well with the observed ZINC Fragments Now (ZFN) median docking scores of the highest ranking 1000 fragments (*R* = −0.717); **(B)** when translated to *in vitro* to either DSF or X-Ray screens using fragment libraries with overlapping chemical space, a similar correlation can be observed, highlighting the suitability of a purely *in silico* druggability workflow as a standalone method (*R* = −0.826); **(C)** DSF fragment screens reported here yielded strong stabilizing fragment hits that are structural subunits of reported ligands for NUDT1 (Gad et al., 2014; Huber et al., 2014).

When SiteMap was applied on the NUDIX hydrolases, the obtained Dscores of the highest-ranking sites were between 0.51 and 1.11 with an average of 0.88 (Figure 5A and Supplementary Material -SiteMap). Interestingly, except for NUDT4, all identified highest-ranking sites were in overlapping regions or even identical with sites identified with DoGSite (Supplementary Material - FTMap). Thus, the returned lower Dscore values for NUDT4 (0.51) and NUDT18 (0.61) were consistent between these approaches. In addition, judging by SiteMap, NUDT3 (0.74), NUDT6 (0.77), NUDT10 (0.59), NUDT20 (0.73), and PTP1B (2HNP, 0.78) were classed as difficult drug targets. The highest-ranking members and thus favored drug targets in the family were NUDT1 (1.02), NUDT5 (1.11) NUDT7 (1.04), NUDT9 (1.01), NUDT12 (1.05), NUDT15 (1.00), NUDT17 (1.01), and NUDT22 (1.04, Figure 3). Due to the chosen cut-off distance to merge identified pockets (5 Å), SiteMap identified large extended pockets which included several subpockets. Furthermore, as NUDT5, NUDT12, and NUDT15 are functional homodimers, these have two high-ranking pockets. Of these, NUDT12 and NUDT15 contain a third druggable site. NUDT7on the other hand possesses a second high-ranking pocket (Dscore 0.82).

**Figure 6:**
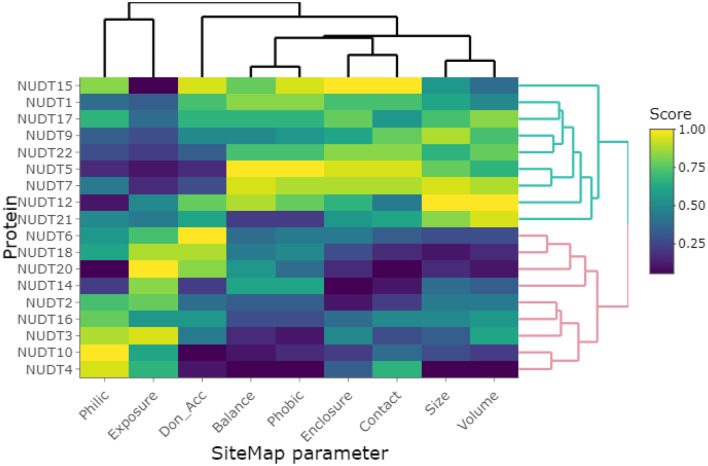
Clustering of investigated NUDIX family members based on the primary SiteMap parameters of the top-ranked sites, resulting in a clear separation of the members deemed druggable (green branches) and those deemed undruggable (red branches).

Clustering of the highest-scoring SiteMap pockets using the primary SiteMap parameters shows a clear separation of druggable versus undruggable NUDIX members and allows for comparison of members which are (dis)similar in terms of their active site properties rather than based on sequence (dis)similarities (Figure 6). Full-length sequence identity is generally low among the NUDIX family members (see Supplementary Material, Percentage Identity), with the exception of NUDT3, NUDT4, NUDT10, and NUDT11. The former 3 being deemed challenging targets and NUDT11 was not evaluated due to lack of structural data. General selectivity issues are thus not anticipated when targeting a specific NUDIX family member. On the other hand, several members have some degree of overlap in their substrate specificity, e.g. NUDT1, 15 and 18 as a subgroup, and NUDT5, NUDT9, NUDT12, and NUDT14 as a second subgroup (Carreras-Puigvert et al., 2017), implying that their active sites share some structural similarity. In this context, the SiteMap parameter profile of NUDT5 is a good reference as it has the highest Dscore of all members. In comparison, NUDT9 has a less favorable balance in hydrophobic and hydrophilic character, while the active site of NUDT12 is somewhat more exposed than NUDT5, but also larger. Although these 3 members have high Dscores, classing them clearly as druggable, they vary in their capacity to accommodate different fragments, as it is reflected by their different median docking scores further down (Figure 5). NUDT14 is considered challenging, primarily due to its smaller active site which also is more exposed.

NUDT15 and NUDT18 have been hypothesized to be able to act as back-up enzymes for NUDT1 due to their overlapping substrate specificities. Comparison of their SiteMap parameter profiles shows clear differences despite NUDT1 and NUDT15 being among the NUDIX members with highest Dscores. The active site of NUDT15 is somewhat smaller and more enclosed than for NUDT1 due to an inward movement of a helix (Carter et al., 2015). NUDT18 is considered to be challenging due to its small and more exposed active site. This also results in a poor fragment scoring profile (please see below). Collectively, these differences in site parameters allow for the development of highly selective chemical probes, as witnessed for NUDT1 and NUDT5 (Gad et al., 2014; Page et al., 2018).

### User Arm – Step 2: *in silico* Docking of ZINC Library

As a final druggability assessment and potential to identify starting points amenable for a fragment growing-based drug discovery campaign out of commercial fragment space, we performed *in silico* docking campaigns of the ZINC Frag now database (Irwin et al., 2012) against the structures under consideration. The comprehensive fragment library was filtered against unwanted structural motifs and prepared for docking using a KNIME workflow (Berthold et al., 2008). For a detailed description, please refer to the Methods part of this manuscript. Ultimately, 205,891 fragments remained after filtering the original set of 704,041 ZINC fragments. Using the Schrödinger suite, ligand preparation and grid generation for the highest-ranking pocket as identified by SiteMap were performed to enable virtual screening of this subset applying three stages of accuracy. In each stage, the top-ranked 10% of compounds were retained and passed on to the next stage. Finally, the top-ranked 1,000 fragments were used to calculate a median docking score enabling assessment of druggability based on commercially available fragment space. The returned median docking scores, where lower is better, ranged from−4.0 to −11.4 kcal/mol with an average of−6.8 kcal/mol. NUDT1 and NUDT5, both validated drug targets in the literature, scored−11.4 and −9.9 kcal/mol respectively. In addition, and judged by the median docking score, NUDT17 (−8.8 kcal/mol) is a third promising drug target. PTP1B (−6.8 kcal/mol) scores average among the NUDIX family members, while the scores for NUDT4 (−4.0 kcal/mol), NUDT10 (−4.3 kcal/mol), NUDT20 (−4.7 kcal/mol) and NUDT18 (−5.0 kcal/mol) indicate a potentially challenging drug discovery campaign (Figure 5A). When using the median docking scores and plotted against their respective SiteMap Dscores, a good inverse correlation (*R* = −0.717, Bravais-Pearson) can be observed (Figure 5), suggesting an *in silico*-based prioritization scheme of drug discovery campaigns against NUDIX proteins. Thus, fragment docking against the top-ranked SiteMap pockets recapitulates their druggability potential but additionally provides potential starting points readily accessible for fragment-based drug discovery campaigns.

The hydrophobicity of small-molecule drugs is a property which needs to be delicately balanced since it affects multiple parameters including solubility, permeability, plasma protein binding and metabolism. Druggable binding pockets of target proteins therefore require a certain hydrophobic-hydrophilic balance to accommodate ligands with drug-like properties. When applying a balance of at least 0.5 the SiteMap assessment prefers NUDT1 (3Q93, 0.69), NUDT5 (6GRU, 1.34), NUDT7 (5T3P, 0.72), NUDT15 (5BON, 0.62), NUDT17 (5LF8, 0.50), and NUDT22 (5LF9, 0.58) and disfavors NUDT3 (2FVV, 0.01), NUDT4 (5LTU, 0.00), NUDT10 (3MCF, 0.01), and PTP1B (2HNP, 0.05). With regard to their returned median fragment docking scores, pocket polarity might correlate with either higher or lower scores (Supplementary Material – ZINC fragment docking and SiteMap). A possible explanation is, that the library was filtered to fit a drug-like profile and thus preselects for druggable proteins itself, ignoring their respective pocket properties. Importantly, none of the crystal structures used here were bound to high-affinity lead compounds originating from drug discovery campaigns and hence no hydrophobic subpockets induced by such compounds where probed in this study.

When combined, the top-1,000 ranked fragments obtained for the 18 protein targets comprised 13,203 unique fragments, indicating a certain amount of “promiscuity,” i.e., fragments binding to 2 or more proteins (36% of fragments). In fact, 73 fragments bound to 6 or more targets (see Supplementary Material - Fragment promiscuity), with one fragment hitting 11 out of 18 proteins. It should be noted that the average docking scores were rather poor, ranging from −7.56 to −5.53 kcal/mol. Of interest is the notion that the proteins deemed undruggable by SiteMap appeared to be enriched for promiscuous fragment hits (except NUDT10 and NUDT20), as opposed to druggable proteins (except NUDT15 and NUDT22). A certain degree of promiscuity should be expected when docking 200K fragments to multiple targets, as this is in line with the basic concept of fragment-based drug discovery, i.e., the ability of low-complexity fragments to interact with a multitude of (sub)pockets across a wide range of proteins.

### Correlation With Experimental Fragment-Based and Biochemical Screening Data

**Figure 7:**
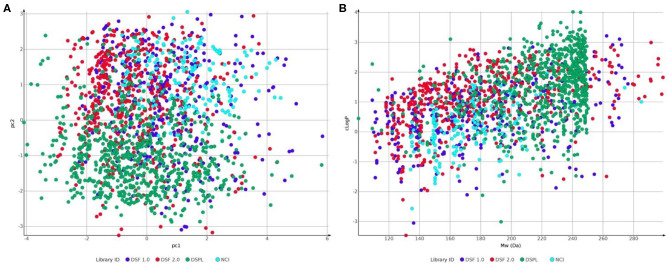
Comparison of fragment libraries used in screens against NUDIX protein family members: Laboratory for Chemical Biology at Karolinska Institutet (LCBKI) DSF fragment library of the first generation (DSF 1.0, blue), additional members of a second generation DSF fragment library (DSF 2.0, red), Nucleobase analogs (NCI, light blue) and Diamond-SGC Poised Library (DSPL, green) with respect to: **(A)** physicochemical property coverage and diversity, expressed as the first two principal components (pc1 and pc2) obtained from a principal component analysis (PCA) on six Lipinski-type properties; **(B)** clogP against Molecular weight.

A number of fragment screens against NUDIX proteins have been performed by others and us^1^. For a list of applied screening techniques, library sets and hit rates, please see Table 2. When the hit rates of the fragment screens were compared with the *in silico*-derived median ZFN docking scores a good correlation was observed (Bravais-Pearson 0.826; Figure 5B). This underscores the applicability of *in silico* docking for rapid protein druggability assessment. In agreement with most computational assessments, NUDT1 and NUDT5 yield high hit rates of 9.8% and 14.7%, respectively, while the experimental hit rate of 0.9% for NUDT4 confirms its challenging character predicted by computational assessment. Other NUDIX proteins are in the range of common hit rates for fragment screens and between 1.6 and 5.6% (Aretz et al., 2014). This observation holds true for different sets screened by different groups (Figures 5B, 7). Interestingly, the DSF screen against NUDT1 found two structures with a strong thermal stabilization of 5°C. These structures are fragments of the reported NUDT1 inhibitors TH588 (IC50: 2.1 nM) and Crizotinib (IC50: 48 nM) and thus underscore the suitability of DSF to find starting points for lead generation (Figure 5C). However, DSF is not feasible for proteins with high native melting points (e.g., NUDT5, 76°C), and here *in silico* fragment screening against druggable sites may be particularly advantageous.

**Table 2:** Summary of NUDIX protein family members in screens against fragment and in biochemical malachite green assays.

**Protein**	**Screening technique**	**Size screening set**	**Hit rate**
NUDT7	Fragment covalent (Resnick et al., 2019)	993	36 (3.6%)
NUDT4	Fragment diamond Xray ^1^	768	7 (0.9%)
NUDT5	Fragment diamond Xray ^1^	768	113 (14.7%)
NUDT7	Fragment diamond Xray ^1^	768	39 (5.1%)
NUDT21	Fragment diamond Xray ^1^	768	43 (5.6%)
NUDT22	Fragment diamond Xray ^1^	768	12 (1.6%)
NUDT1	Fragment DSF	450	44 (9.8%)
NUDT2	Fragment DSF	650	35 (5.3%)
NUDT5	Fragment DSF	450	0^*^
NUDT15	Fragment DSF	1,200	60 (5.0%)
NUDT1	Biochem screen malachite green	5,336	429 (8.0%)
NUDT2	Biochem screen malachite green	5,336 [11,992]	261 (4.9%) [235 (2.0%)^**^]
NUDT5	Biochem screen malachite green	5,336 [72,004]	3 (0.1%) [527 (0.7%)^**^]
NUDT15	Biochem screen malachite green	5,336 [17,908]	10 (0.2%) [98 (0.5%)^**^]
NUDT16	Biochem screen malachite green	5,336	7 (0.1%)

*Please note the biochemical screen library grew over time. For comparison, results beyond the basic set of 5,336 compounds are presented in brackets*.

* *Melting temperature unsuitable for thermal shift screens*;

** *Definition of hit more stringent*.

Several biochemical screening campaigns against NUDIX proteins have also been performed in our laboratories. While compound libraries have varied somewhat between targets, reflecting development of the compound libraries over time, there is a small core set of about 5,300 chemically diverse compounds that have been tested for all proteins. It is noteworthy that these screens were performed based on a common screening platform employing a coupled enzymatic assay with a malachite green readout. This cost-effective assay has been frequently employed in our lab, including screens on other nucleotide-processing targets such as dCTPase (Llona-Minguez et al., 2016), ITPase and dUTPase, and with robust performance in compound sets beyond 100,000 compounds (all unpublished). A key reason for this is the low rates of interference with the coupled enzymes and the absorbance readout at 630 nm, as evidenced by a low appearance of common hits. Also, the presence of PAINS and aggregators within hit lists is generally low for this family of proteins (Supplementary Material – Screens using malachite green), demonstrating robust screening performance of the recombinantly produced proteins and other assay components. The biochemical screen outcomes are summarized in detail in Table 2 and in the Supplementary Material – Screens using malachite green. In line with assessments of chemical amenability and learnings in the fragment-based screens, the majority of targets generated hits that confirmed activity in follow-up studies, with NUDT1 demonstrating an extreme hit rate in this sub-set. This significant amenability is in line with the publication of hits from multiple groups. A critical outlier in this set was NUDT5, which demonstrated hit rates as low as notoriously challenging targets dUTPase and ITPase, while predictions and fragment-based screening showed the opposite (Supplementary Material – Fragment screening hit rates). Already at the time of screening we had a reason to revisit the screening data for NUDT5 and follow-up studies demonstrated competition between active site hits and a structurally important Mg^2+^ (Costa and Dieckmann, 2011; Vardakou et al., 2014). After correction of the assay buffer, by lowering the MgCl_2_ concentration 10-fold, we observed significantly higher hit rates in the subsequently applied compound sets and identified compounds that could be further optimized to nM potencies (Page et al., 2018). As a general observation and for the five NUDIX protein members screened, no correlation between ZINC fragment docking scores and observed hit rates from biochemical screening can be observed (Supplementary Material – Screens using malachite green). In contrast to the covered fragment library chemical space, a rule-of-five compliant library of few thousand compounds may complement the search for a chemical starting point but may be limited in coverage of chemical space itself. However, in the past we have shown, that embarking on drug discovery campaigns from observed hits in both fragment and biochemical screen lead to successful generation of chemical probes for a number of NUDIX protein family members and other pyrophosphatases (Gad et al., 2014; Llona-Minguez et al., 2016; Page et al., 2018)^2^.

## Conclusion and Summary

Here we presented a dual *in silico* druggability assessment workflow suitable for large-scale evaluation of proteins and protein families, applied to the NUDIX family. Initially, we introduced a hands-on workflow solely based on the protein crystal structure using the open access server of DogSite (Volkamer et al., 2012) and FTMap (Kozakov et al., 2015) for rigid and CryptoSite (Cimermancic et al., 2016) and FTMap for dynamic assessment. Importantly, before using these servers, thorough manual protein structure verification is necessary to exclude artifacts due to crystal packing, construct used and resolution limits. On the one hand, DogSite returns both identity and score of druggable sites, while FTMap docks small organic solvent molecules. Especially in cases of sparsely evaluated proteins or protein complexes this dual assessment may be beneficial for structural assessment and potential chemical probe generation. On the other hand, CryptoSite identifies conformationally active amino acid residues. In the past, the returned cryptic scores have been correlated with FTMap solvent docking and eased decision on whether or where potential allosteric sites may be situated (Vajda et al., 2018). Timewise, this quick computational assessment may be achieved within days for singular proteins and weeks for small protein families. Depending on local load and choice of sever location the return time is usually minutes for DogSite, hours for FTMap, and 1 day for CryptoSite. The detailed assessment and correlation of data from the different algorithms allows for the rationalization of targeting strategies. In case of the NUDIX proteins, NUDT22 for example showed to have high scores in DogSite and FTMap with CryptoSite confirming flexibility around two closely related sites. Further, in the past we have shown that comparing different crystals structures of the same protein can allow for the observation of targetable conformations more suitable small-molecule development (Michel et al., 2019). With the open access deposition of all screening data by the SGC, a similar albeit more time consuming approach is possible for a number of NUDIX proteins^1^.

In a second, user-guided arm we assessed protein druggability employing several implemented functions in Schrödinger's commercial small-molecule modeling suite combined with freely available KNIME (Berthold et al., 2008). First, proteins were interrogated for potential binding pockets and the corresponding DScores using SiteMap. The highest ranking pockets were then used to perform cascade docking with a filtered ZINC fragment library (Irwin et al., 2012). Subsequently, the median docking score of the top-1,000 ranked fragments was used as a chemical space-based druggability assessment. Both parameters, DScore and median docking score form the basis of this second *in silico* druggability assessment and require few days per protein, depending on the user set up and the size of the used *in silico* library. The observed docking scores correlate well with predicted DScores (Figure 5A) and additionally provide commercially accessible chemical starting points for the development of chemical probes. At last, when compared with experimental fragment screens based on X-ray crystallography, a covalent set and thermal stabilization in DSF, a similar correlation was observed between hit rates and median docking scores (Figure 5B), even when using chemically distinct screening sets (Figure 7). This supports the applicability of an *in silico* druggability workflow as a standalone method for protein assessment and speaks for the chemical space coverage of fragment libraries generated at CBCS and Diamond/SGC (Michel et al., 2019)^7^.

In summary, we report here a fully *in silico* druggability assessment of the NUDIX protein family, that serves as a standalone method and a workflow to identify the most suitable members for a drug discovery campaign. We show that the dual assessment correlates well with experimental results and further allows for the *in silico* identification of secondary druggable sites, alternative targeting strategies and structural basis for fragment growing campaigns. Importantly, the workflow allows for rapid assessment of any protein with reported structures in the protein data bank and as such should be broadly applicable in early drug discovery campaigns.

## Data Availability Statement

All datasets generated for this study are included in the article/Supplementary Material.

## Author Contributions

MM and EH compiled the list of PDB structures and performed protein preparation and validation. MM controlled end stage correlation and assessment and performed tasks in the automated arm. EH performed tasks in the User arm. EW, KP, IA, A-LG, and TL performed assays, fragment, and biochemical high-throughput screens. MM and EH rationalized and designed the project. UW and TH supervised the project. MM, EH, AG, and TL wrote the manuscript. MM coordinated collaborations. All authors commented on the manuscript.

## Conflict of Interest

TH is listed as an inventor of patents describing NUDT1 and NUDT5 inhibitors. EH is listed as an inventor of a patent describing NUDT1 inhibitors. The patents are fully owned by a non-profit public foundation, the Helleday Foundation, and TH and UW are member of the foundation board developing inhibitors toward and in the clinic (NCT03036228). TL is an employee of AstraZeneca, but performed all experimental work associated with this publication while at Chemical Biology Consortium Sweden. The remaining authors declare that the research was conducted in the absence of any commercial or financial relationships that could be construed as a potential conflict of interest.

## Footnotes

^1^*Fragment Screening*. Available online at: https://www.thesgc.org/fragment-screening (accessed August 8, 2019).

^2^NUDT15. *Manuscript Submitted*.

^3^*Zentrum für Bioinformatik: Universität Hamburg - Proteins Plus Server*. Available online at: https://proteins.plus/ (accessed June 3, 2019).

^4^*Cryptic binding site*. Available online at: https://modbase.compbio.ucsf.edu/cryptosite/ (accessed June 3, 2019).

^5^*FTMap: A Small Molecule Mapping Server*. Available online at: http://ftmap.bu.edu/login.php (accessed June 3, 2019).

^6^*Introduction to Heatmaply*. Available online at: https://cran.r-project.org/web/packages/heatmaply/vignettes/heatmaply.html (accessed April 16, 2020).

^7^*DSI-Poised Library - MX - Diamond Light Source*. Available online at: https://www.diamond.ac.uk/Instruments/Mx/Fragment-Screening/Fragment-Libraries/DSI-poised-library.html (accessed October 7, 2019).

## References

[B1] Abdelraheim S. R. Spiller D. G. McLennan A. G. (2003). Mammalian NADH diphosphatases of the nudix family: cloning and characterization of the human peroxisomal NUDT12 protein. Biochem. J. 374(Pt. 2), 329–335. 10.1042/bj20030441 12790796 PMC1223609

[B2] An S. Fu L. (2018). Small-molecule PROTACs: an emerging and promising approach for the development of targeted therapy drugs. EBioMedicine 36, 553–562. 10.1016/j.ebiom.2018.09.005 30224312 PMC6197674

[B3] Aretz J. Anumala U. R. Fuchsberger F. F. Molavi N. Ziebart N. Zhang H.. (2018). Allosteric inhibition of a mammalian lectin. J. Am. Chem. Soc. 140, 14915–14925. 10.1021/jacs.8b08644 30303367

[B4] Aretz J. Wamhoff E.-C. Hanske J. Heymann D. Rademacher C. (2014). Computational and experimental prediction of human C-type lectin receptor druggability. Front. Immunol. 5:323. 10.3389/fimmu.2014.00323 25071783 PMC4090677

[B5] Baell J. B. Holloway G. A. (2010). New substructure filters for removal of pan assay interference compounds (PAINS) from screening libraries and for their exclusion in bioassays. J. Med. Chem. 53, 2719–2740. 10.1021/jm901137j 20131845

[B6] Barford D. Flint A. J. Tonks N. K. (1994). Crystal structure of human protein tyrosine phosphatase 1B. Science 263, 1397–1404. 10.1126/science.8128219 8128219

[B7] Baykov A. A. Evtushenko O. A. Avaeva S. M. (1988). A malachite green procedure for orthophosphate determination and its use in alkaline phosphatase-based enzyme immunoassay. Anal. Biochem. 171, 266–270. 10.1016/0003-2697(88)90484-8 3044186

[B8] Berthold M. R. Cebron N. Dill F. Gabriel T. R. Kötter T. Meinl T. (2008). “KNIME: The Konstanz Information Miner,” in Data Analysis, Machine Learning and Applications; Studies in Classification, Data Analysis, and Knowledge Organization, eds Preisach C. Burkhardt H. Schmidt-Thieme L. Decker R. (Berlin; Heidelberg: Springer). p. 319–326. 10.1007/978-3-540-78246-9_38

[B9] Caffrey J. J. Hidaka K. Matsuda M. Hirata M. Shears S. B. (1999). The human and rat forms of multiple inositol polyphosphate phosphatase: functional homology with a histidine acid phosphatase up-regulated during endochondral ossification. FEBS Lett. 442, 99–104. 10.1016/S0014-5793(98)01636-6 9923613

[B10] Caffrey J. J. Safrany S. T. Yang X. Shears S. B. (2000). Discovery of molecular and catalytic diversity among human diphosphoinositol-polyphosphate phosphohydrolases. an expanding nudt family. J. Biol. Chem. 275, 12730–12736. 10.1074/jbc.275.17.12730 10777568

[B11] Carreras-Puigvert J. Zitnik M. Jemth A.-S. Carter M. Unterlass J. E. Hallström B.. (2017). A comprehensive structural, biochemical and biological profiling of the human NUDIX hydrolase family. Nat. Commun. 8:1541. 10.1038/s41467-017-01642-w 29142246 PMC5688067

[B12] Carter M. Jemth A.-S. Hagenkort A. Page B. D. G. Gustafsson R. Griese J. J.. (2015). Crystal structure, biochemical and cellular activities demonstrate separate functions of MTH1 and MTH2. Nat. Commun. 6:7871. 10.1038/ncomms8871 26238318 PMC4532830

[B13] Choi S.-Y. Jang J. H. Kim K. R. (2011). Analysis of Differentially Expressed Genes in Human rectal carcinoma using suppression subtractive hybridization. Clin. Exp. Med. 11, 219–226. 10.1007/s10238-010-0130-5 21331762

[B14] Cimermancic P. Weinkam P. Rettenmaier T. J. Bichmann L. Keedy D. A. Woldeyes R. A.. (2016). CryptoSite: expanding the druggable proteome by characterization and prediction of cryptic binding sites. J. Mol. Biol. 428, 709–719. 10.1016/j.jmb.2016.01.029 26854760 PMC4794384

[B15] Coseno M. Martin G. Berger C. Gilmartin G. Keller W. Doublié S. (2008). Crystal structure of the 25 KDa subunit of human cleavage factor I_m_. Nucleic Acids Res. 36, 3474–3483. 10.1093/nar/gkn079 18445629 PMC2425470

[B16] Costa J. B. D. Dieckmann T. (2011). Entropy and Mg2+ control ligand affinity and specificity in the malachite green binding RNA aptamer. Mol. BioSyst. 7, 2156–2163. 10.1039/c1mb05075c 21523267

[B17] Dubianok Y. Collins P. Krojer T. Fairhead M. MacLean E. Diaz S. (2018). LPDB 6gru Structure Summary ‹ Protein Data Bank in Europe (PDBe) ‹ EMBL-EBI. Available online at: https://www.ebi.ac.uk/pdbe/entry/pdb/6gru (accessed June 3, 2019).

[B18] Ellermann M. Eheim A. Rahm F. Viklund J. Guenther J. Andersson M.. (2017). Novel class of potent and cellularly active inhibitors devalidates MTH1 as broad-spectrum cancer target. ACS Chem. Biol. 12, 1986–1992. 10.1021/acschembio.7b00370 28679043

[B19] Farand J. Kropf J. E. Blomgren P. Xu J. Schmitt A. C. Newby Z. E.. (2020). Discovery of potent and selective MTH1 inhibitors for oncology: enabling rapid target (In)validation. ACS Med. Chem. Lett. 11, 358–364. 10.1021/acsmedchemlett.9b00420 32184970 PMC7074220

[B20] Friesner R. A. Banks J. L. Murphy R. B. Halgren T. A. Klicic J. J. Mainz D. T.. (2004). Glide: a new approach for rapid, accurate docking and scoring. 1. method and assessment of docking accuracy. J. Med. Chem. 47, 1739–1749. 10.1021/jm0306430 15027865

[B21] Friesner R. A. Murphy R. B. Repasky M. P. Frye L. L. Greenwood J. R. Halgren T. A.. (2006). Extra precision glide: docking and scoring incorporating a model of hydrophobic enclosure for protein–ligand complexes. J. Med. Chem. 49, 6177–6196. 10.1021/jm051256o 17034125

[B22] Gad H. Koolmeister T. Jemth A.-S. Eshtad S. Jacques S. A. Ström C. E.. (2014). MTH1 inhibition eradicates cancer by preventing sanitation of the DNTP pool. Nature 508, 215–221. 10.1038/nature13181 24695224

[B23] Ge H. Chen X. Yang W. Niu L. Teng M. (2013). Crystal structure of wild-type and mutant human Ap4A hydrolase. Biochem. Biophys. Res. Commun. 432, 16–21. 10.1016/j.bbrc.2013.01.095 23384440 PMC7092880

[B24] Greenwood J. R. Calkins D. Sullivan A. P. Shelley J. C. (2010). Towards the comprehensive, rapid, and accurate prediction of the favorable tautomeric states of drug-like molecules in aqueous solution. J. Comput. Aided Mol. Des. 24, 591–604. 10.1007/s10822-010-9349-1 20354892

[B25] Halgren T. (2007). New method for fast and accurate binding-site identification and analysis. Chem Biol Drug Des. 69, 146–148. 10.1111/j.1747-0285.2007.00483.x 17381729

[B26] Halgren T. A. (2009). Identifying and characterizing binding sites and assessing druggability. J. Chem. Inf. Model 49, 377–389. 10.1021/ci800324m 19434839

[B27] Halgren T. A. Murphy R. B. Friesner R. A. Beard H. S. Frye L. L. Pollard W. T.. (2004). Glide: a new approach for rapid, accurate docking and scoring. 2. enrichment factors in database screening. J. Med. Chem. 47, 1750–1759. 10.1021/jm030644s 15027866

[B28] Huber K. V. M. Salah E. Radic B. Gridling M. Elkins J. M. Stukalov A.. (2014). Stereospecific targeting of MTH1 by (S)-crizotinib as an anticancer strategy. Nature 508, 222–227. 10.1038/nature13194 24695225 PMC4150021

[B29] Irwin J. J. Sterling T. Mysinger M. M. Bolstad E. S. Coleman R. G. (2012). ZINC: a free tool to discover chemistry for biology. J. Chem. Inf. Model 52, 1757–1768. 10.1021/ci3001277 22587354 PMC3402020

[B30] Kettle J. G. Alwan H. Bista M. Breed J. Davies N. L. Eckersley K.. (2016). Potent and selective inhibitors of MTH1 probe its role in cancer cell survival. J. Med. Chem. 59, 2346–2361. 10.1021/acs.jmedchem.5b01760 26878898

[B31] Kozakov D. Grove L. E. Hall D. R. Bohnuud T. Mottarella S. E. Luo L.. (2015). The FTMap family of web servers for determining and characterizing ligand-binding hot spots of proteins. Nat. Protoc. 10, 733–755. 10.1038/nprot.2015.043 25855957 PMC4762777

[B32] Krishnan N. Konidaris K. F. Gasser G. Tonks N. K. (2018). A potent, selective, and orally bioavailable inhibitor of the protein-tyrosine phosphatase PTP1B improves insulin and leptin signaling in animal models. J. Biol. Chem. 293, 1517–1525. 10.1074/jbc.C117.819110 29217773 PMC5798283

[B33] Llona-Minguez S. Höglund A. Jacques S. A. Johansson L. Calderón-Montaño J. M. Claesson M.. (2016). Discovery of the first potent and selective inhibitors of human DCTP pyrophosphatase 1. J. Med. Chem. 59, 1140–1148. 10.1021/acs.jmedchem.5b01741 26771665 PMC4753678

[B34] Mathea S. Salah E. Velupillai S. Tallant C. Pike A. C. W. Bushell S. R. (2017b). PDB 5mp0 Structure Summary ‹ Protein Data Bank in Europe (PDBe) ‹ EMBL-EBI. Available online at: https://www.ebi.ac.uk/pdbe/entry/pdb/5mp0 (accessed June 3, 2019).

[B35] Mathea S. Tallant C. Salah E. Wang D. Velupillai S. Nowak R. (2017a). PDB 5lf8 Structure Summary ‹ Protein Data Bank in Europe (PDBe) ‹ EMBL-EBI. Available online at: https://www.ebi.ac.uk/pdbe/entry/pdb/5lf8 (accessed June 3, 2019).

[B36] Michel M. Visnes T. Homan E. J. Seashore-Ludlow B. Hedenström M. Wiita E.. (2019). Computational and experimental druggability assessment of human DNA glycosylases. ACS Omega 4, 11642–11656. 10.1021/acsomega.9b00162 31460271 PMC6682003

[B37] Mullard A. (2018). Phosphatases start shedding their stigma of undruggability. Nat. Rev. Drug Discov. 17, 847–849. 10.1038/nrd.2018.201 30482950

[B38] Niesen F. H. Berglund H. Vedadi M. (2007). The use of differential scanning fluorimetry to detect ligand interactions that promote protein stability. Nat. Protoc. 2, 2212–2221. 10.1038/nprot.2007.321 17853878

[B39] Page B. D. G. Valerie N. C. K. Wright R. H. G. Wallner O. Isaksson R. Carter M.. (2018). Targeted NUDT5 inhibitors block hormone signaling in breast cancer cells. Nat. Commun. 9:250. 10.1038/s41467-017-02293-7 29343827 PMC5772648

[B40] Petrocchi A. Leo E. Reyna N. J. Hamilton M. M. Shi X. Parker C. A.. (2016). Identification of potent and selective MTH1 inhibitors. Bioorg. Med. Chem. Lett. 26, 1503–1507. 10.1016/j.bmcl.2016.02.026 26898335

[B41] Resnick E. Bradley A. Gan J. Douangamath A. Krojer T. Sethi R.. (2019). Rapid covalent-probe discovery by electrophile-fragment screening. J. Am. Chem. Soc. 141:8951–8968. 10.1021/jacs.9b02822 31060360 PMC6556873

[B42] Samaranayake G. J. Huynh M. Rai P. (2017). MTH1 as a chemotherapeutic target: the elephant in the room. Cancers 9:47. 10.3390/cancers9050047 28481306 PMC5447957

[B43] Shelley J. C. Cholleti A. Frye L. L. Greenwood J. R. Timlin M. R. Uchimaya M. (2007). Epik: a software program for PK prediction and protonation state generation for drug-like molecules. J. Comput. Aided. Mol. Des. 21, 681–691. 10.1007/s10822-007-9133-z 17899391

[B44] Shen B. W. Perraud A.-L. Scharenberg A. Stoddard B. L. (2003). The crystal structure and mutational analysis of human NUDT9. J. Mol. Biol. 332, 385–398. 10.1016/S0022-2836(03)00954-9 12948489

[B45] Srikannathasan V. Nunez C. A. Tallant C. Siejka P. Mathea S. Kopec J. (2017b). PDB 5t3p Structure Summary ‹ Protein Data Bank in Europe (PDBe) ‹ EMBL-EBI. Available online at: https://www.ebi.ac.uk/pdbe/entry/pdb/5t3p (accessed June 3, 2019).

[B46] Srikannathasan V. Nunez C. A. Tallant C. Siejka P. Mathea S. Newman J. (2017a). PDB 5ltu Structure Summary ‹ Protein Data Bank in Europe (PDBe) ‹ EMBL-EBI. Available online at: https://www.ebi.ac.uk/pdbe/entry/pdb/5ltu (accessed June 3, 2019).

[B47] Tallant C. Siejka P. Mathea S. Shrestha L. Krojer T. Srikannathasan V. (2017). PDB 5lf9 structure summary ‹ Protein Data Bank in Europe (PDBe) ‹ EMBL-EBI. Available online at: https://www.ebi.ac.uk/pdbe/entry/pdb/5lf9 (accessed June 3, 2019).

[B48] Thorsell A.-G. Persson C. Gräslund S. Hammarström M. Busam R. D. Hallberg B. M. (2009). Crystal structure of human diphosphoinositol phosphatase 1. Proteins 77, 242–246. 10.1002/prot.22489 19585659

[B49] Trésaugues L. Lundbäck T. Welin M. Flodin S. Nyman T. Silvander C.. (2015). Structural basis for the specificity of human nudt16 and its regulation by inosine monophosphate. PLoS ONE 10:e0131507. 10.1371/journal.pone.0131507 26121039 PMC4485890

[B50] Tresaugues L. Moche M. Arrowsmith C. H. Berglund H. Bountra C. Collinsm R. (2009a). PDB 3h95 Structure Summary ‹ Protein Data Bank in Europe (PDBe) ‹ EMBL-EBI. Available online at: https://www.ebi.ac.uk/pdbe/entry/pdb/3h95 (accessed June 3, 2019).

[B51] Tresaugues L. Moche M. Arrowsmith C. H. Berglund H. Busam R. D. Collins R. (2008). PDB 3cou Structure Summary ‹ Protein Data Bank in Europe (PDBe) ‹ EMBL-EBI. Available online at: https://www.ebi.ac.uk/pdbe/entry/pdb/3cou (accessed June 3, 2019).

[B52] Tresaugues L. Siponen M. I. Arrowsmith C. H. Berglund H. Bountra C. Collins R. (2011a). PDB 3q93 structure summary ‹ Protein Data Bank in Europe (PDBe) ‹ EMBL-EBI. Available online at: https://www.ebi.ac.uk/pdbe/entry/pdb/3q93 (accessed June 3, 2019).

[B53] Tresaugues L. Siponen M. I. Arrowsmith C. H. Berglund H. Bountra C. Collins R. (2011b). PDB 3q91 Structure Summary ‹ Protein Data Bank in Europe (PDBe) ‹ EMBL-EBI. Available online at: https://www.ebi.ac.uk/pdbe/entry/pdb/3q91 (accessed June 3, 2019).

[B54] Tresaugues L. Siponen M. I. Lehtio L. Arrowsmith C. H. Berglund H. Bountra C. (2009b). PDB 3gg6 Structure Summary ‹ Protein Data Bank in Europe (PDBe) ‹ EMBL-EBI. Available online at: https://www.ebi.ac.uk/pdbe/entry/pdb/3gg6 (accessed June 3, 2019).

[B55] Tresaugues L. Welin M. Arrowsmith C. H. Berglund H. Bountra C. Collins R. (2010). PDB 3mcf structure summary ‹ Protein Data Bank in Europe (PDBe) ‹ EMBL-EBI. Available online at: https://www.ebi.ac.uk/pdbe/entry/pdb/3mcf (accessed June 3, 2019).

[B56] Tsuzuki T. Egashira A. Kura S. (2001). Analysis of MTH1 gene function in mice with targeted mutagenesis. Mutat. Res. 477, 71–78. 10.1016/S0027-5107(01)00108-7 11376688

[B57] Vajda S. Beglov D. Wakefield A. E. Egbert M. Whitty A. (2018). Cryptic binding sites on proteins: definition, detection, and druggability. Curr. Opin. Chem. Biol. 44, 1–8. 10.1016/j.cbpa.2018.05.003 29800865 PMC6088748

[B58] Vardakou M. Salmon M. Faraldos J. A. O'Maille P. E. (2014). Comparative analysis and validation of the malachite green assay for the high throughput biochemical characterization of terpene synthases. MethodsX 1, 187–196. 10.1016/j.mex.2014.08.007 26150952 PMC4472957

[B59] Volkamer A. Kuhn D. Rippmann F. Rarey M. (2012). DoGSiteScorer: a web server for automatic binding site prediction, analysis and druggability assessment. Bioinformatics 28, 2074–2075. 10.1093/bioinformatics/bts310 22628523

[B60] Walters W. P. Murcko M. A. (2002). Prediction of “drug-likeness”. Adv. Drug Deliv. Rev. 54, 255–271. 10.1016/S0169-409X(02)00003-0 11922947

[B61] Warpman Berglund U. Sanjiv K. Gad H. Kalderén C. Koolmeister T. Pham T.. (2016). Validation and development of MTH1 inhibitors for treatment of cancer. Ann. Oncol. 27, 2275–2283. 10.1093/annonc/mdw429 27827301

[B62] Wenthur C. J. Gentry P. R. Mathews T. P. Lindsley C. W. (2014). Drugs for allosteric sites on receptors. Annu Rev Pharmacol Toxicol 54, 165–184. 10.1146/annurev-pharmtox-010611-134525 24111540 PMC4063350

[B63] Wright R. H. G. Lioutas A. Le Dily F. Soronellas D. Pohl A. Bonet J.. (2016). ADP-ribose-derived nuclear ATP synthesis by NUDIX5 is required for chromatin remodeling. Science 352, 1221–1225. 10.1126/science.aad9335 27257257

[B64] Wu H. Li L. Chen K.-M. Homolka D. Gos P. Fleury-Olela F.. (2019). Decapping enzyme NUDT12 partners with BLMH for cytoplasmic surveillance of NAD-capped RNAs. Cell Rep. 29, 4422–4434.e13. 10.1016/j.celrep.2019.11.108 31875550

[B65] Yueh C. Rettenmaier T. J. Xia B. Hall D. R. Alekseenko A. Porter K. A.. (2019). Kinase atlas: druggability analysis of potential allosteric sites in kinases. J. Med. Chem. 62, 6512–6524. 10.1021/acs.jmedchem.9b00089 31274316 PMC7019049

[B66] Zhang S. Zhang Z.-Y. (2007). PTP1B as a drug target: recent developments in PTP1B inhibitor discovery. Drug Discov. Today 12, 373–381. 10.1016/j.drudis.2007.03.011 17467573

[B67] Zhou W. Ma L. Yang J. Qiao H. Li L. Guo Q.. (2019). Potent and specific MTH1 inhibitors targeting gastric cancer. Cell Death Dis. 10:434. 10.1038/s41419-019-1665-3 31164636 PMC6547740

